# Smart transplants: emerging role of nanotechnology and big data in kidney and islet transplantation, a frontier in precision medicine

**DOI:** 10.3389/fimmu.2025.1567685

**Published:** 2025-04-08

**Authors:** Rajkiran Deshpande, Titus Augustine

**Affiliations:** ^1^ Department of Renal and Pancreas Transplantation and General Surgery, Manchester Royal Infirmary, Manchester University Foundation Trust, Manchester, United Kingdom; ^2^ Department Faculty of Biology, Medicine and Health, Division of Diabetes, Endocrinology and Gastroenterology, Manchester Academic Health Science Centre, University of Manchester, Manchester, United Kingdom

**Keywords:** nanotechnology, big data, kidney transplantation, islet transplantation, precision medicine, artificial intelligence

## Abstract

Kidney and islet transplantation has revolutionized the management of renal failure and diabetes. Transplantation is considered as excellent therapeutic intervention for most suitable patients. While advancements in the surgical aspects, immunosuppression and outcomes have potentially plateaued, new technologies have developed which could enhance transplantation with benefits to patients and clinical teams alike. The science of nanotechnology and big data advancements are two such technologies, collectively paving the way for smarter transplantation solutions. Nanotechnology offers novel strategies to overcome critical challenges, including organ preservation, ischemia-reperfusion injury and immune modulation. Innovations such as nanoparticle-based drug delivery systems, biocompatible encapsulation technologies for islet transplants, and implantable artificial kidneys are redefining the standards of care. Meanwhile, big data analytics harness vast datasets to optimize donor-recipient matching, refine predictive models for post-transplant outcomes, and personalize therapeutic regimens. Integrating these technologies forms a synergistic framework where nanotechnology enhances therapeutic precision and big data provides actionable insights, enabling clinicians to adopt proactive, patient-specific strategies. By addressing unmet needs and leveraging the combined potential of nanotechnology and big data, this transformative approach promises to improve graft survival, functionality, and overall patient outcomes, marking a paradigm shift in transplantation medicine. These developments will also be accelerated with integration of the rapidly advancing science of artificial intelligence.

## 1 Introduction

Organ transplantation is a vital treatment for patients with end-stage organ failure, significantly improving survival and quality of life. Advances in surgery, anesthesia, and immunosuppression have enhanced graft survival and patient outcomes. However, the rising prevalence of chronic illnesses and increasing life expectancy have driven organ demand far beyond available supply, creating a critical shortage (1–3). This shortage, combined with risks such as immune rejection and the side effects of immunosuppressive drugs, limits the broader application of transplantation (2, 4–6). Techniques such as single-cell sequencing, which provides insights into cell heterogeneity and immune rejection markers, and CRISPR/Cas9 gene-editing technology, which facilitates the creation of transgenic pigs to address organ shortages, are reshaping the landscape of transplantation medicine (7–9). The integration of artificial intelligence solutions is set to change the face of healthcare and transplantation.

Nanotechnology, in particular, has become a transformative tool in transplantation. Nanomaterials, typically ranging from 1 to 100 nanometers (nm), offer unique properties for diagnosing and treating diseases at a molecular level (10). Applications include targeted drug delivery (5–10), improved pharmacokinetics (11), reduced toxicity (12, 13), tumour treatment (14), and advanced molecular imaging (15). In transplantation, nanotechnology addresses key challenges such as optimizing immunosuppressive drug delivery, enhancing organ preservation, advancing artificial organ development, and improving imaging techniques (5, 16),. The integration of big data further enhances transplantation outcomes by enabling better donor-recipient matching, predicting complications, and optimizing postoperative care. Combining nanotechnology with predictive analytics can revolutionize patient management, tackling issues of organ shortages and improving long-term outcomes.

## 2 Methodology

In this mini review, a focused synthesis of the literature on nanotechnology, big data, and artificial intelligence (AI) in islet and kidney transplantation was conducted. Given the scope of this review, an exhaustive analysis of all available databases and studies was not undertaken. A systematic search was performed using PubMed, ScienceDirect, and Scopus, utilizing keywords such as “nanotechnology in islet and kidney transplantation,” “big data in organ transplantation,” “kidney transplantation advancements,” “nanomaterials in organ preservation,” and “AI in transplantation.” Articles published between 1994 and 2025 were included. The selection criteria were defined to ensure the relevance and quality of the included studies. Peer-reviewed articles, original research, and systematic reviews published in English that focused on applications of nanotechnology, big data, or AI in organ transplantation were included. In contrast, articles were excluded if they were not directly relevant to organ transplantation, were limited to conference abstracts, editorials, or opinion pieces lacking substantial data, or exhibited insufficient methodological transparency. Furthermore, no formal bias or reliability assessment tools were employed to evaluate the quality of the studies included. To mitigate selection bias, literature from a broad range of journals and sources were reviewed, incorporating both supportive and critical perspectives on AI, big data and nanotechnology in transplantation. However, the potential for publication bias remains a recognized limitation, as studies reporting favorable AI outcomes are more likely to be published. This review acknowledges these challenges and aims to provide a balanced perspective by discussing both the advantages and limitations of big data, nanotechnology and AI-driven approaches in organ transplantation.

## 3 Nanotechnology in kidney and islet cell transplantation

Understanding how nanomaterials behave in the kidneys is crucial for their effective use in medical applications. The physical and chemical properties of the nanomaterials, such as size, shape, charge, and surface ligands, influence this behavior. Smaller particles, particularly those under 5 nm, can pass through the glomerular filtration barrier (GFB) and be excreted in the urine. Particles smaller than 1 nm may even accumulate in the endothelial glycocalyx of the glomerulus due to enhanced interactions with its structure. Larger nanomaterials, on the other hand, are usually excluded by the GFB and tend to accumulate in other renal compartments. For example, nanomaterials smaller than 100 nm cannot pass the GFB and often collect in the mesangium. At the same time, larger particles are absorbed by endothelial cells in the peritubular capillaries and secreted into the proximal tubules. This size-dependent behavior is essential for designing nanomaterials that target specific kidney regions. By adjusting these nanomaterial’s size, shape, and surface properties, researchers can optimize their therapeutic effects, ensuring they are delivered to the right area while minimizing side effects or unwanted accumulation (17).

### 3.1 Nanotechnology in the preservation of donor kidneys

Preservation of donor kidneys is critical, especially for donation after circulatory death (DCD) donors. The current standard, static cold storage, reduces oxygen demand by cooling the organ, but its limitations are significant. With a preservation window of only 24–36 hours, it increases the risk of ischemia-reperfusion injury (IRI), particularly in extended criteria donors who are more prone to acute and chronic rejection (18, 19). Ex vivo machine perfusion techniques such as hypothermic machine perfusion (HMP) and normothermic machine perfusion (NMP) have emerged to address these challenges. HMP, which is already established in clinical practice, uses cold temperatures with controlled perfusion to mitigate IRI, inflammation, and delayed graft function. Meanwhile, NMP, which supplies oxygen and nutrients under physiological conditions to sustain metabolism and reduce ischemic injury, is gaining increasing interest within the transplant community. NMP also enables kidney assessment and potential repair before transplantation, with ongoing studies evaluating its broader clinical application (20). Given the good results obtained in animal trials, NMP has shown promise in preclinical and early clinical trials, including its use in select cases in the United kingdom to successfully transplant kidneys previously deemed unsuitable (21). Large-scale clinical trials are still ongoing to establish its definitive clinical benefit. Furthermore, strategies involving therapeutic interventions during perfusion, such as drug and molecular treatments, are currently being evaluated in experimental and preclinical models (21).

Several preclinical trials are evaluating the use of nano-agents in Ex vivo perfusion systems, which provide a platform for delivering targeted nanotherapeutic agents directly to the kidney. For instance, Zhang et al. developed amphiphilic chitosan micelles loaded with the acetaldehyde dehydrogenase 2 agonist alda-1. These micelles, introduced during HMP, reduced ischemic injury through antibacterial and antioxidant effects, improving graft quality (22). Similarly, Tietjen et al. conjugated nanoparticles with anti-CD31 antibodies to specifically target endothelial cells during NMP. This approach enhanced nanoparticle delivery to poorly perfused areas, significantly improving graft quality and reducing rejection risks (21). Another innovation is nano artificial oxygen carriers. Traditional red blood cell-based carriers face challenges such as availability, mismatches, and infection risks. Jagers et al. developed 4% albumin-derived nanocapsules containing perfluorocarbons (PFCs). These nanocapsules reduced hypoxia and improved renal function during NMP by mitigating markers like hypoxia-inducible factors 1α and 2α (23, 24). Additionally, extracellular vesicles (EVs)—naturally occurring nanoparticles released by cells—are emerging biomarkers for real-time graft assessment during perfusion. For example, Woud et al. used imaging flow cytometry and nanoparticle tracking analysis to identify EV subsets in perfusates. They found that CD9+ EVs correlated with cold ischemia time, while CD31+ EVs were linked to renal blood flow and vascular resistance, providing valuable insights into graft viability (25, 26).

### 3.2 Implantable artificial kidneys

Kidney transplantation is the gold standard treatment for end-stage renal disease (ESRD), restoring kidney function and improving quality of life. However, the shortage of donor organs forces many patients to rely on dialysis, which, while life-sustaining, comes with significant lifestyle limitations and long-term health risks. IAK is a promising alternative that integrates silicon nanotechnology with tissue engineering to mimic natural kidney functions, offering a potential solution to both transplantation and traditional dialysis (27, 28). IAK comprises two main components designed to replicate kidney filtration and processing. The HemoCartridge filters blood using silicon nanotechnology. Microchips create filtration channels resembling glomerular slit diaphragms, allowing waste molecules to pass while retaining essential substances like proteins. Blood pressure powers this process, eliminating the need for external pumps or dialysate. The second component, the BioCartridge, functions as a bioreactor containing cultured renal tubular epithelial cells. It processes the ultrafiltrate produced by the HemoCartridge, reabsorbing critical substances like glucose, salts, and water while concentrating toxins into a small fluid volume similar to urine (29).

The IAK’s design prioritizes biocompatibility. Silicon membranes are coated with hydrated polymers to minimize blood stagnation and shear stress, reducing clotting risks without anticoagulants. The HemoCartridge employs slit-pore nanotechnology to simulate filtration, while the BioCartridge uses living tubular cells to process filtrate. The device connects directly to the patient’s bloodstream (like iliac vessels), relying solely on blood pressure for function, simplifying its design (29). While the IAK has been proposed to offer several advantages over dialysis, these remain theoretical at this stage. In concept, it could eliminate the need for dialysate, as the BioCartridge is designed to reabsorb essential substances to maintain fluid balance. Additionally, using the patient’s renal cells aims to enhance compatibility, potentially reducing immune rejection and the need for immunosuppressants. However, these benefits have not yet been fully achieved in clinical practice. The IAK remains in early experimental stages, and further research is required to determine its feasibility and effectiveness in reducing dependence on donor kidneys and improving the quality of life for dialysis patients (27–29). However, several challenges have hindered its progression to clinical application. The device relies on a blood pressure gradient for hemofiltration, necessitating implantation in major vascular structures, which may limit future kidney transplant options. Additionally, achieving effective ultrafiltration remains a significant hurdle, and the bioreactor may require periodic replacement due to the limited longevity of cultured renal tubular cells (30).The development of IAK is a collaborative effort involving academic institutions, with preclinical testing and device miniaturization currently underway. Efforts to sustain renal epithelial cell function, including cryopreservation through Bioartificial Renal Epithelial Cell Systems (BRECS), have shown some promise in preclinical models (30). Despite these advancements, the device has yet to enter clinical trials and further research is needed to address these technical and physiological challenges, safety issues, efficacy, and feasibility before clinical translation. If these hurdles are overcome, future trials could pave the way for FDA approval and eventual clinical integration (28, 30).

### 3.3 Nanotechnology in islet transplantation

Type 1 diabetes mellitus (T1DM) is an autoimmune disease that destroys pancreatic β-cells, impairing insulin secretion and poor glycemic control (31). While exogenous insulin therapy helps manage blood glucose levels, it has limitations such as injection site pain, inflammation, and the risk of hypoglycemia (32). Islet transplantation offers a less invasive alternative to pancreas transplantation, restoring physiological insulin secretion and endogenous glycemic control (33, 34). This procedure infuses purified cadaveric human islets into the hepatic portal vein. The Edmonton Protocol, introduced in 2000, revolutionized clinical islet transplantation (CIT) with a steroid-free immunosuppression regimen and a standardized islet graft mass of 10,000 islet equivalents (IEQ) per kilogram of body weight (35, 36). This approach significantly improved glycemic control and reduced reliance on exogenous insulin in patients with severe T1DM (37). However, widespread adoption of CIT faces challenges, including a limited availability of pancreatic islet donors compared to the magnitude off diabetes and number of eligible patients, the need for lifelong immunosuppression, poor cell engraftment, and recurrent autoimmunity (34, 37–40). These issues limit its long-term efficacy and suitability for broader patient populations. Nanotechnology offers innovative solutions to enhance the effectiveness and durability of islet transplantation. Nanoscale engineering techniques enable precise localization and controlled distribution of therapeutic agents, improving cellular engraftment by providing immunoprotection and potentially reducing lifelong immunosuppression (41, 42). Additionally, targeted delivery systems optimized with nanotechnology can potentially enhance treatment outcomes while minimizing side effects (42).

#### 3.3.1 Nanoscale immunoisolation and microencapsulation

Microencapsulation of islets using semipermeable membranes is an innovative strategy for achieving transplantation without needing immunosuppressive agents (43, 44). This method involves encapsulating pancreatic islets within a protective barrier that prevents direct contact with immune cells while allowing the exchange of essential nutrients and waste products. Lim and Sun pioneered the concept of islet encapsulation, first using sodium alginate to encase pancreatic islets. Their approach effectively shielded the islets from immune system components without requiring immunosuppressive agents, thereby mitigating immune responses while maintaining their ability to uptake nutrients (45). Subsequent research has focused on optimizing the materials and methods for encapsulation to enhance its efficacy. These advancements include improving control over the thickness of the capsule walls and the size of the microcapsules, enhancing the durability of the capsules, and reducing the toxicity of the materials used. Such refinements have significantly improved the viability and functionality of encapsulated islets of Langerhans (45, 46). Despite these advancements, challenges remain. While encapsulation prevents direct immune reactions, issues with biocompatibility persist. The body’s nonspecific foreign body reaction to the microcapsules can lead to fibrous tissue proliferation around the capsules. This fibrotic response can impair the exchange of nutrients and oxygen between the islets and their external environment, ultimately causing necrosis of the encapsulated islets (47).

#### 3.3.2 Reducing immunoinflammatory response around microcapsules

Anti-inflammatory drugs can help mitigate fibroproliferation around microcapsules, but the effectiveness of various drugs remains uncertain, and systemic administration of these drugs can lead to adverse reactions. Dang et al. conducted an intravital subcutaneous screening of 16 anti-inflammatory drugs to explore potential solutions. Their findings revealed that dexamethasone and curcumin were the most effective in inhibiting early inflammatory proteases and reactive oxygen species (ROS). Based on these results, they proposed co-encapsulating these drugs with islets in alginate microcapsules. The localized release of the drug within the microcapsules was found to inhibit fibrosis around them, thereby enhancing the blood glucose control of the microencapsulated islets (47). Similarly, Azadi et al. developed an alginate/dextran-spermine microcapsule that encapsulated both pancreatic islets and pentoxifylline (PTX), an anti-inflammatory drug. This microcapsule effectively protected the islets, alleviated the surrounding inflammatory response, and reduced fibroproliferation. Their approach demonstrated that localized anti-inflammatory treatment could significantly improve outcomes of microencapsulated islet transplantation (48).

#### 3.3.3 Layer-by-layer polymer self-assembly for improved immunoprotection

While microencapsulation has proven effective in protecting islets, challenges remain due to the large volume of microcapsules required. Excessive volume can interfere with islet metabolic processes and limit transplant site options, often necessitating less optimal sites like the peritoneal cavity, which has lower blood supply. This increases the risk of graft failure due to hypoxia, highlighting the need for more refined encapsulation strategy. LBL polymer self-assembly presents a promising solution by creating a thin nano-sized film coating, which can reduce the size of encapsulated islets and provide adequate immune protection. Wilson et al. demonstrated that LBL self-assembly using a poly(L-lysine)-g-poly(ethylene glycol) (biotin) (PPB) and streptavidin (SA) coating successfully protected islets without affecting their viability, function, or nutrient exchange. This approach allows for smaller islet volumes, enabling transplantation to more desirable sites, such as the portal vein, which were previously restricted due to microcapsule size (49–51). Haque et al. validated the feasibility of LBL encapsulation in a non-human primate model by applying three layers of poly (ethylene glycol) (PEG) to the surface of islets. The LBL-coated islets showed reduced immunogenicity, maintained function and viability, and had a higher survival rate than non-coated islets. Additionally, these islets suffered less immune damage when treated with glucocorticoid-free immunosuppressive regimen, demonstrating the potential of LBL technology for transplantation (52). Kizilel developed a biofunctional multilayer film using LBL to immobilize glucagon-like peptide-1 (GLP-1) on the surface of islets to enhance its function. This coating improved islet survival and promoted insulin secretion without affecting the response time to glucose. This method could reduce the number of islets required for transplantation, addressing the shortage of donor islets while minimizing the risk of elevated portal pressure after transplantation (53).

## 4 Big data in enhancing organ transplantation

Big data refers to large, complex datasets that are difficult to manage with traditional tools. In organ transplantation, big data analytics can improve clinical outcomes, optimize processes, and foster personalized care by integrating diverse sources like electronic health records (EHRs), genomics, imaging, biomarkers, and administrative databases. Leveraging artificial intelligence (AI), machine learning (ML), and computational tools, big data addresses critical challenges such as organ shortages, graft rejection, and patient care optimization.

### 4.1 Improving donor-recipient matching through big data

Big data has revolutionized donor-recipient matching by refining organ allocation. Traditional donor allocation models such as kidney donor profile index (KDPI), model of end-stage liver disease (MELD), kidney allocation system (KAS), and lung allocation system (LAS) rely on predefined scoring systems that assess organ viability and recipient survival likelihood (54, 55). AI-driven models like optimal prediction of mortality (OPOM) (56), artificial neural networks (ANN)-based systems (55), and random survival forest (RSF) (57) enhance donor-recipient matching by integrating ML techniques that analyze large datasets, including biomarkers and patient demographics. However, AI-driven systems face several challenges compared to traditional models. They lack transparency and interpretability, making it difficult for clinicians to understand or justify allocation decisions (58). Unlike established scoring systems with predefined parameters, algorithmic bias and data limitations may result in disparities in organ allocation, particularly for underrepresented patient groups (59). AI models depend on historical data, which can reinforce existing inequities if the training datasets are not representative. Additionally, these systems require extensive validation across diverse populations before implementation in clinical practice (60). Furthermore, AI-based models demand significant computational resources and continuous updates, which may not be feasible in all transplant centers, especially in resource-limited settings. Ethical concerns regarding autonomy, fairness, and accountability further complicate AI’s integration into transplantation (59). Given these limitations, hybrid approaches, combining traditional scoring systems with AI-driven models, may provide an optimal balance between transparency, fairness, and precision in organ allocation (61–64). **Table 1** briefly outlines key differences between traditional donor allocation models and AI-driven models.

**Table 1:** Comparison of traditional and AI-based donor allocation models in organ transplantation.

Feature	Traditional Models	AI-Based Models
Criteria considered	Clinical variables like age, comorbidities, HLA (human leucocyte antigen) matching (61)	Multivariate analysis incorporating biomarkers, genomics, and real-time patient data (57)
Decision-making process	Fixed scoring systems like MELD, KDPI and KAS (54, 55)	Adaptive learning models that evolve with more data (56)
Predictive accuracy	Based on predefined parameters, requiring periodic updates (57)	AI models like OPOM, ANN, and RSF predict survival and graft function more precisely (56, 57)
Efficiency	Manual evaluation, time-consuming prioritization (58)	Automated, rapid assessment ensuring real-time prioritization (58)
Bias & limitations	Transparent and limited by predefined parameters (54).	Potential algorithmic bias, requiring validation and explainability (59)
Clinical integration	Well-established, widely accepted (60)	Needs further validation for full adoption (60)
Ethical concerns	Follows predefined allocation rules, ensuring fairness and accountability (59, 60)	Raises concerns about autonomy, fairness, and accountability, especially in decision-making without human oversight (59).

### 4.2 Predicting graft survival and rejection using ML

The application of big data analytics in transplant holds promise for improving graft and patient survival predictions. ML also plays a key role in predicting graft survival and detecting rejection. By analyzing from biomarkers, genomics, and clinical histories, ML models can help identify risk factors for graft rejection and failure (59). ML models when trained on big data analytics have the potential to help predict patient outcomes and enable early intervention (65). It can risk stratify and improve long-term graft survival predictions. ML and statistical models are increasingly used to predict renal graft survival, balancing accuracy with clinical interpretability. Model selection plays a crucial role, as it should balance prediction performance with clinical interpretability, depending on the use context (65). Key prognostic predictors include acute and chronic rejection episodes, post-transplant urological complications, patient nonadherence, elevated blood urea nitrogen (BUN) levels, and regular physical exercise. Acute and chronic rejection episodes remain among the most critical determinants of graft survival, reinforcing the need for close immunosuppressive management. Post-transplant complications, including urinary tract infections and ureteral obstructions, also significantly impact long-term outcomes (65).

Furthermore, patient adherence to medication and follow-up care plays a crucial role (65). While ML models have demonstrated promise in risk stratification, their integration into clinical practice remains an ongoing challenge. Most studies focus on retrospective analyses, and external validation in diverse patient populations is still required before these models can be widely adopted. Incorporating big data, including EHRs and multi-omics data, may enhance predictive accuracy and enable personalized medicine approaches. Future efforts should focus on validating these models in real-world settings and optimizing their usability for transplant clinicians (65).

### 4.3 Optimizing treatment protocols via large-scale data

Large-scale datasets, including those from biobanks and EHRs, are crucial for evaluating treatment effectiveness and refining clinical protocols. By analyzing data from global centers, researchers can uncover trends and associations which help optimize immunosuppressive regimens, surgical methods, and post-operative care. Real-time monitoring of transplant outcomes through these datasets also helps identify disparities in care, leading to policy changes that promote equitable access to quality transplantation services. Integrating big data analytics, predictive modelling, and large-scale data analysis transforms organ transplantation. As technology continues to evolve, it holds the promise of reducing organ wastage, enhancing long-term transplant outcomes, and advancing personalized medicine in transplantation (64, 66–68).

### 4.4 Challenges in big data and nanotechnology for organ transplantation

Big data and artificial intelligence (AI) have emerged as transformative tools in organ transplantation, promising to revolutionize donor-recipient matching, predictive analytics, and personalized medicine. However, their integration into clinical practice is fraught with significant challenges that must be addressed to ensure these technologies are safe, ethical, and effective.

#### 4.4.1 Data availability and bias

One of the most significant barriers to utilizing big data in transplantation is the lack of comprehensive, representative datasets. While expansive, current data repositories, such as Medicare claims databases, the Scientific Registry of Transplant Recipients, and clinical trial databases, are incomplete and often fail to capture the nuances of diverse patient populations. This limitation leads to concerns about bias and generalizability in AI algorithms. Training datasets may underrepresent complex cases, comorbidities, or patients undergoing novel treatments, precisely the cohorts most likely to benefit from personalized recommendations. Additionally, missing or inconsistent data can result in suboptimal algorithm performance and inaccurate predictions. For example, local variations in clinical practice may create discrepancies between training and real-world data, compromising the reliability of AI-driven predictions (69, 70). Addressing these issues requires creating more inclusive datasets that are validated rigorously. Clinicians must also be trained to interpret AI predictions critically and identify when a model might fail, ensuring its integration adds value rather than harm to clinical decision-making.

#### 4.4.2 Decision process and AI explainability

The “black box” nature of many AI algorithms, such as neural networks and random forests, presents another challenge. Unlike simpler models like decision trees, these complex algorithms often lack interpretability, making it difficult for clinicians to understand how recommendations are derived. This lack of transparency can lead to scepticism or misuse, especially when AI predictions conflict with clinical judgment or fail to provide actionable insights (71, 72). Developing interpretable AI models and sensitivity analysis techniques can help mitigate this issue. However, these solutions often add complexity, introducing new layers of uncertainty. Furthermore, research shows that explaining AI recommendations may not constantly improve decision-making and can sometimes lead to over-reliance on flawed AI outputs (73).

#### 4.4.3 Ethical and regulatory considerations

The acceptability of AI in clinical practice raises ethical concerns. Questions such as “When is AI good enough to use?” and “How do we ethically incorporate AI recommendations into patient care?” highlight the need for robust ethical guidelines and standardized accuracy benchmarks. Regulatory frameworks, such as the FDA’s Software as a Medical Device (SaMD) protocol, aim to ensure commercial AI tools undergo rigorous evaluation. However, these protocols do not always apply to in-house AI tools developed by hospitals for research purposes, creating potential discrepancies in quality and safety. Transparency in AI development, evaluation, and deployment is critical to building trust and ensuring accountability (74).

#### 4.4.4 Limited clinical translation

Nanotechnology presents another frontier in transplantation, offering novel solutions for organ preservation, ischemia-reperfusion injury (IRI), and immunosuppression. However, translating nanotechnology from experimental settings to clinical practice remains challenging due to the lack of standardized safety assessments and the need for optimizing biocompatibility. The nanomaterials necessitates rigorous toxicity and safety evaluations to determine their long-term effects in transplant patients. Additionally, understanding how nanomaterials interact with biological systems—including their clearance rates and potential side effects—is crucial for their safe integration into transplantation protocols. Regulatory frameworks must evolve to accommodate nanotechnology-based interventions, ensuring they meet safety and efficacy standards while maintaining feasibility for widespread clinical use.

### 4.5 Future directions

AI and nanotechnology are poised to revolutionize organ transplantation by improving donor-recipient matching, optimizing surgical planning, enhancing diagnostics, and advancing targeted therapies. However, their widespread clinical integration requires large-scale, multi-centre trials to validate safety, efficacy, and long-term benefits. Future AI-driven transplantation studies should focus on integrating ML with genomic, proteomic, and metabolomic data to refine donor-recipient matching. Precision compatibility assessments based on multi-omics profiling could reduce immunological risks and rejection rates and improve long-term graft survival (75, 76). Additionally, AI models should be further trained to detect rejection markers from histopathological biopsy images, incorporating automated scoring systems to support early diagnosis and treatment planning. Surgical applications of AI should be explored through advanced imaging analysis, real-time intraoperative decision support, and AI-assisted robotic surgery platforms. ML algorithms can refine preoperative risk assessments, enhance organ viability assessments, and provide predictive analytics for surgical complications. AI-guided imaging analysis could assist in islet transplantation by improving pancreatic islet quantification and viability assessment, facilitating better graft outcomes (77, 78). Future research should also investigate AI’s role in automating perfusion-based organ assessments during ex vivo normothermic perfusion, helping optimize transplant suitability. Ensuring fairness and reliability in AI models is critical. Biases arising from non-representative datasets must be addressed through fairness-aware ML approaches. Incorporating diverse patient data, continuous model validation across demographic subgroups, and explainability-focused AI models will be key to fostering trust and equity in AI-driven transplantation (79).

Nanotechnology, with its potential to enhance organ preservation, mitigate ischemia-reperfusion injury (IRI), and deliver targeted immunosuppression, remains a promising but underutilized tool. Future research must prioritize clinical trials assessing nanomaterial stability, biodegradation, and immunogenicity to ensure biocompatibility and safety. Additionally, nanoparticle-based drug delivery systems could enable personalized immunosuppressive therapies, reducing systemic toxicity and enhancing localized therapeutic effects. Trials should also investigate how nanotechnology-enhanced organ preservation solutions, such as oxygen-carrying nanoparticles or antioxidant-loaded nanosystems, can improve graft viability and extend preservation times. Finally, regulatory and ethical frameworks must evolve alongside these technological advancements. Establishing standardized methodologies for AI validation and nanotechnology approval is essential to facilitate their transition from preclinical research to routine clinical practice. Close collaboration between clinicians, regulatory agencies, and technology developers will be crucial in ensuring these innovations’ safe, effective, and equitable implementation in transplantation.

## 5 Conclusion

Nanotechnology, AI and big data are revolutionizing kidney and islet transplantation by addressing critical challenges such as organ preservation, ischemia-reperfusion injury and immunosuppression. Nanomaterial-based preservation improves organ viability, while biocompatible encapsulation enhances islet survival and function. Integrating it with AI-driven predictive models refines donor-recipient matching and post-transplant care, allowing early organ rejection or dysfunction management. Although these advances are yet to be transitioned to clinical practice, they will pave the way for precision transplantation medicine and improved patient outcomes. Sir Peter Medawar aptly stated, “The transplantation of tissues and organs will one day be a routine practice, but its success depends on bridging science with clinical application.” These advancements pave the way for precision transplantation medicine and improved patient outcomes.
